# *Aspergillus niger* otomycosis in a child with chronic otitis externa

**DOI:** 10.4102/sajid.v35i1.128

**Published:** 2020-06-25

**Authors:** Christoffel J. Opperman, Julie Copelyn

**Affiliations:** 1 Division of Medical Microbiology, National Health Laboratory Service, University of Cape Town and Groote Schuur Hospital, Cape Town, South Africa; 2 Department of Paediatrics and Child Health, Faculty of Health Sciences, University of Cape Town, Cape Town, South Africa

**Keywords:** *Aspergillus niger*, otomycosis, chronic otitis externa, otorrhea, antifungal

## Abstract

*Aspergillus niger* is commonly associated with otomycosis. Currently, local guidelines lack appropriate guidance on the definitive treatment and management when the fungus is identified. The repeated use of antibiotics in children with refractory episodes of otitis externa is concerning and may contribute towards otomycosis. This case report highlights the importance of a well-collected pus swab from the ear and suggests a topical antifungal to use in paediatric patients with Aspergillus niger otomycosis.

## Case presentation and management

A 5-year-old girl was referred to the ear, nose and throat (ENT) clinic at a tertiary hospital with a 5-month history of right ear otalgia and foul smelling otorrhea. Symptoms had not resolved, despite two courses of oral co-amoxiclav received from a private general practitioner and local clinic, as well as a 6-week course of ciprofloxacin ear drops prescribed as three drops 8-hourly. The child was otherwise healthy with no previous history of significant illness. Screening for human immunodeficiency virus was negative. On clinical examination the patient was apyrexial and systemically well. No nasal obstruction or discharge was noted. Otoscopic examination revealed blood and pus in the right external auditory canal, from which a pus swab was sent from the outpatient department. The patient did not tolerate aural toilet in the clinic and was thus booked for examination under anaesthesia in theatre. Findings in theatre included an intact tympanic membrane on the right with prominent pus, debris, and a cotton-wool-like foreign body in the external auditory canal. The left ear had only wax visible. Both ears were cleaned in theatre, but no specimen sent to the laboratory. The child was discharged on ciprofloxacin ear drops for another 2 weeks, for a total treatment duration of 8 weeks of the fluroquinolone. A follow-up visit was booked at the ENT outpatient clinic in 6 weeks’ time. On review the discharge was reported as resolved with no specific treatment of the *Aspergillus niger* cultured during the initial clinic visit. Grommets were inserted 2 months later.

## Laboratory identification of *Aspergillus niger*

Pus swabs are routinely inoculated onto 4% blood agar (anaerobic conditions), boiled blood agar (carbon dioxide conditions) and MacConkey media (aerobic conditions), incubated at 35 °C for the isolation of bacteria and non-filamentous fungi. However, if a fungus is suspected and requested for culturing on the laboratory form, additional media will be inoculated to enhance the growth of a fungus. This includes Sabouraud Dextrose (SabDex) media with amikacin, incubated at 35 °C and 25 °C, as well as brain heart infusion agar incubated aerobically at 35 °C. Furthermore, plates will be incubated for up to 3 weeks to monitor growth and up until 6 weeks if dimorphic fungi are suspected.

**FIGURE 1:**
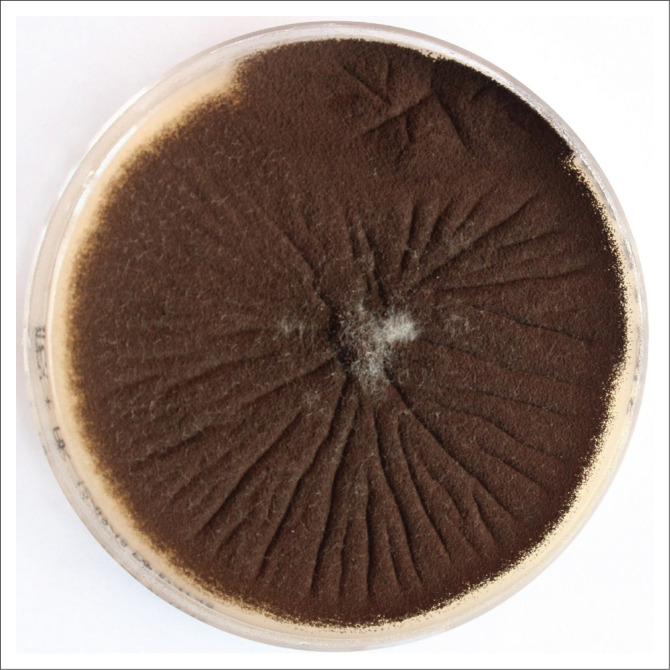
Characteristic black sporing heads of *Aspergillus niger* covering white mycelium macroscopically, grown at 35° C on Sabouraud Dextrose media with amikacin.

**FIGURE 2:**
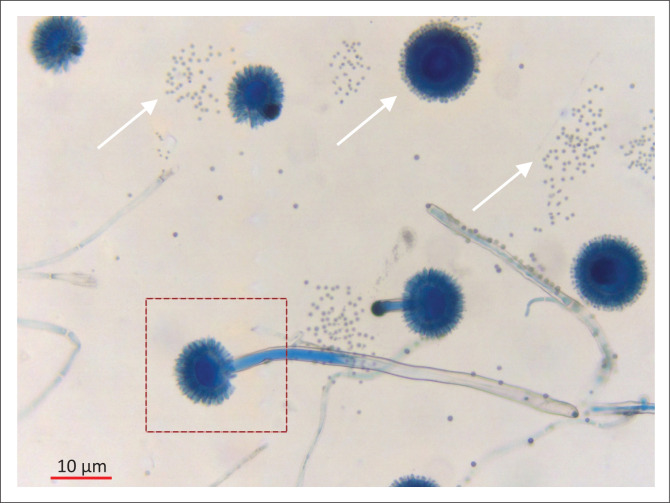
Microscopic view of *Aspergillus niger* stained with lactophenol cotton blue (binocular, light microscope: 200X). The arrows indicate roughened conidia. The box area highlights the conidiophore with a large vesicle, as well as the phialides and metulae covering the entire surface.

In this case *Aspergillus niger* was grown at 25 °C and 35 °C on SabDex media with amikacin from the pus swab taken from the outpatient department. Macroscopic appearance was of a flat surface with radial folds and a granular texture with characteristic black sporing heads covering white mycelium (Figure 1). The reverse of the fungus on the culture media plate had a cream colour. Microscopy of a lactophenol cotton blue stained impression showed thick walled conidiophores which had colourless stalks and were smooth with a round vesicle, phialides and metulae covering the entire surface. The conidia itself were round to oval, 5 µm – 10 µm and roughened (Figure 2).

Antifungal susceptibility testing is not offered for any topical agents (clotrimazole) in South Africa. In addition, no breakpoints are available for the interpretation of minimum inhibitory concentrations (MIC) of topical antifungal agents. It is likely that the delivery of an antifungal agent directly to the site of an infection will result in a very high concentration which is probably much higher than the MIC.

### Ethical consideration

This article followed all ethical standards for carrying out research without direct contact with human or animal subjects.

## Discussion

Schwartz et al.^1^ published on the estimated burden of fungal disease in South Africa with minimal data on superficial infections such as otomycosis.^1^ To our knowledge, no recent literature is available on *Aspergillus niger* fungal infections associated with otomycosis in South Africa.

Otorrhoea is a common paediatric presentation, and the majority of cases can be successfully treated with dry mopping of the ear and topical acetic acid or antimicrobial eardrops. Local guidelines do not promote routine investigations in uncomplicated otitis media or otitis externa. However, in refractory cases a well-collected ear swab may assist in providing aetiological diagnosis of chronic otorrhea.

Otomycosis refers to a superficial mycotic infection involving the external ear with the auditory meatus mostly affected.^2^ *Aspergillus* and *Candida* species are the most common causative organisms isolated in patients with otomycosis. Previous studies have found the prevalence of otomycosis worldwide to be around 9% – 30% in patients presenting with otitis externa.^3^ Predisposing factors include the presence of cerumen, increased use of topical antibiotics and steroids, a humid climate, instrumentation and self-cleaning of the ear with foreign objects, an immunocompromised host, open-cavity mastoidectomy surgery, the use of hearing aids with occlusive ear mold and the accumulation of epithelial debris in the external auditory canal.^3,4^ Interestingly, otomycosis is more prevalent in the 20- to 30-year age group than in children 10 years and younger.^5^

Common presenting symptoms include unilateral otalgia, persistent otorrhea, pruritus and tinnitus. Additionally, patients may report progressive hearing loss secondary to fungal debris in the ear canal. Otoscope examination may reveal a black fluffy growth in the ear canal when *Aspergillus niger* is present. It is not uncommon for otomycosis to develop in patients following acute bacterial otitis media with otorrhea.^2,3,4^ This may be exacerbated by the prescription of broad-spectrum antibiotics such as fluoroquinolone eardrops. Whilst fluoroquinolones have been shown to be a safe and effective treatment for otitis media, fungal infections are a well-known complication of broad-spectrum antibacterial use. Furthermore, the discontinuation of antibacterial therapy is central to the treatment of otomycosis.^6^

No consensus exists on the most effective agent for the treatment of otomycosis with only a few publications on its treatment with topical antifungals. It is important to note that some of these studies, as reported by Vennewald and Klemm^2^ do not report on clinical or mycology details, cure rates or administration routes. Most studies include small numbers of patients in observational studies.^2^ Nevertheless, all agree on the importance of local mechanical debridement of visible fungal elements, topical or systemic antifungal agents and the recovery of skin integrity.^2,3,4,5,6^ Topical 1% clotrimazole cream applied twice daily for 2 weeks provides an attractive option for non-invasive infections.^2,5,7^ Clotrimzole shows good efficacy against the *Aspergillus* species, low ototoxicity, greater compliance, lower cost compared to systemic antifungals, is routinely available in peripheral settings and is suitable for application in paediatric patients. Systemic antifungals, especially the triazoles (voriconazole, itraconazole, posaconazole) are reserved for fungal cases of mastoiditis or cerebral mycosis with the guidance of an infectious diseases specialist or microbiologist as an inpatient.^2^

## Conclusion

The correct diagnosis and treatment of otomycosis requires a high level of suspicion in refractory cases of otorrhea. Routinely, pus swabs from the auditory meatus are not recommended as it yields polymicrobial overgrowth. However, in the case of chronic otorrhea or if a clinical suspicion is present of fungal growth, a well-taken pus swab can be very valuable. Clotrimazole cream is an option for the treatment of non-invasive cases of *Aspergillus niger* otomycosis in conjunction with cleaning of the ear canal. Finally, invasive cases should be discussed with a microbiologist or infectious diseases specialist for systemic therapy.
